# Alarming Levels of Drug-Resistant Tuberculosis in HIV-Infected Patients in Metropolitan Mumbai, India

**DOI:** 10.1371/journal.pone.0110461

**Published:** 2014-10-21

**Authors:** Petros Isaakidis, Mrinalini Das, Ajay M V Kumar, Christopher Peskett, Minni Khetarpal, Arun Bamne, Balkrishna Adsul, Mamta Manglani, Kuldeep Singh Sachdeva, Malik Parmar, Avinash Kanchar, B.B. Rewari, Alaka Deshpande, Camilla Rodrigues, Anjali Shetty, Lorraine Rebello, Peter Saranchuk

**Affiliations:** 1 Médecins Sans Frontières, Mumbai, India; 2 International Union Against Tuberculosis and Lung Disease (The Union), South-East Asia Regional Office, New Delhi, India; 3 City TB Office, Bawala Wadi, Chinckpokli, Mumbai, India; 4 Brihanmumbai Municipal Corporation, Mumbai, India; 5 Mumbai District AIDS Control Society (MDACS), Mumbai, India; 6 Pediatric Centre of Excellence for HIV Care, L.T.M. Medical College & General Hospital, Sion, Mumbai, India; 7 Central TB Division, Directorate General of Health Services, Ministry of Health and Family Welfare, New Delhi, India; 8 World Health Organisation - Country Office for India, New Delhi, India; 9 Department of AIDS Control, National AIDS Control Organisation, New Delhi, India; 10 MGM Institute of Health Sciences, Navi Mumbai, India; 11 P.D. Hinduja National Hospital and Medical Research Centre, Mumbai, India; 12 Southern Africa Medical Unit (SAMU), Médecins Sans Frontières, Cape Town, South Africa; University of Delhi, India

## Abstract

**Background:**

Drug-resistant tuberculosis (DR-TB) is a looming threat to tuberculosis control in India. However, no countrywide prevalence data are available. The burden of DR-TB in HIV-co-infected patients is likewise unknown. Undiagnosed and untreated DR-TB among HIV-infected patients is a major cause of mortality and morbidity. We aimed to assess the prevalence of DR-TB (defined as resistance to any anti-TB drug) in patients attending public antiretroviral treatment (ART) centers in greater metropolitan Mumbai, India.

**Methods:**

A cross-sectional survey was conducted among adults and children ART-center attendees. Smear microscopy, culture and drug-susceptibility-testing (DST) against all first and second-line TB-drugs using phenotypic liquid culture (MGIT) were conducted on all presumptive tuberculosis patients. Analyses were performed to determine DR-TB prevalence and resistance patterns separately for new and previously treated, culture-positive TB-cases.

**Results:**

Between March 2013 and January 2014, ART-center attendees were screened during 14135 visits, of whom 1724 had presumptive TB. Of 1724 attendees, 72 (4%) were smear-positive and 202 (12%) had a positive culture for *Mycobacterium tuberculosis*. Overall DR-TB was diagnosed in 68 (34%, 95% CI: 27%–40%) TB-patients. The proportions of DR-TB were 25% (29/114) and 44% (39/88) among new and previously treated cases respectively. The patterns of DR-TB were: 21% mono-resistant, 12% poly-resistant, 38% multidrug-resistant (MDR-TB), 21% pre-extensively-drug-resistant (MDR-TB plus resistance to either a fluoroquinolone or second-line injectable), 6% extensively drug-resistant (XDR-TB) and 2% extremely drug-resistant TB (XDR-TB plus resistance to any group-IV/V drug). Only previous history of TB was significantly associated with the diagnosis of DR-TB in multivariate models.

**Conclusion:**

The burden of DR-TB among HIV-infected patients attending public ART-centers in Mumbai was alarmingly high, likely representing ongoing transmission in the community and health facilities. These data highlight the need to promptly diagnose drug-resistance among all HIV-infected patients by systematically offering access to first and second-line DST to all patients with ‘presumptive TB’ rather than ‘presumptive DR-TB’ and tailor the treatment regimen based on the resistance patterns.

## Introduction

India is a high burden country for tuberculosis (TB) and multidrug-resistant TB (MDR-TB). The World Health Organization has estimated that India accounted for 26% of the total number of TB cases worldwide in 2012, with 2.2% and 15% of the new and retreatment cases respectively being caused by multidrug-resistant strains [1]. Further, India is home to approximately 2.4 million people living with HIV [2] and considered to have a high burden on account of the large absolute numbers of people living with HIV in the country.

The dual burden of HIV and TB/DR-TB in India is significantly high with a combined rate of 5.2%, ranging from 0.4% to 28.8% in various studies, with increasing trends noted in states having a higher burden of HIV infection [3]–[7]. However, nation-wide studies do not exist and previous studies have occurred mainly in hospitals and tertiary care centres [2], [6]–[11]. A crude estimate from these studies suggests that 2500–3000 HIV-infected persons develop MDR-TB annually in India.

Country-wide or state-wide drug resistance surveys (DRS) aim to estimate the DR-TB burden at the country or state level. While this approach is scientifically and operationally acceptable, it may mask significant and important variance in the magnitude of the epidemic in different localities, communities or specific populations. For India, a vast country with an enormous burden of TB and a relatively large burden of HIV in absolute numbers, this statement seems to hold true; from an overcrowded impoverished slum in Mumbai to a small isolated village in the Northern Eastern Provinces of the country, one can assume that several different epidemics may exist. A description of such local epidemics is necessary so as to complement the country-wide prevalence estimate. While there is an urgent need for a nationally representative, country-wide DRS in India, specific studies to identify pockets of extremely high DR-TB prevalence or extensive drug resistance patterns are equally needed in order to advocate for and implement effective control strategies.

The overall aim of this study was to assess the burden of drug-susceptible and drug-resistant tuberculosis among HIV-infected patients attending antiretroviral treatment (ART) centers in the metropolitan area of Mumbai. The specific objectives were a) to determine the proportion of HIV-infected patients with DR-TB among those attending public ART centers, b) to describe drug susceptibility patterns among *Mycobacterium tuberculosis* isolates from this population, and c) to identify factors associated with TB and drug-resistant TB among HIV patients. We aimed to contribute to the evidence base that informs policies and practices and help to estimate the resources needed to control the epidemic in this specific group, as well as the community.

## Methods

### Ethics

The study was approved by the Institutional Ethics Committee of Grant Medical College and Sir J.J. Group of Hospitals (Mumbai, India), the Ethics Review Board of Médecins Sans Frontières (Geneva, Switzerland) and the Ethics Advisory Group of the International Union Against Tuberculosis and Lung Disease (Paris, France). The study protocol was approved by the Indian Council of Medical Research (ICMR), New Delhi, India. Informed consent was obtained from all study participants.

### Study design

This was a cross-sectional survey among HIV-infected adult and paediatric patients attending public and public-private ART clinics in the greater metropolitan Mumbai area. All patients with presumptive pulmonary or extra-pulmonary TB were assessed with smear microscopy and conventional liquid culture. All M. tuberculosis isolates underwent drug susceptibility testing (DST) for first- and second-line anti-TB drugs.

### Sample size

The desired sample size was determined separately for new and previously treated culture positive TB cases. Previous tuberculosis treatment was defined as any anti-tuberculosis treatment reported by the patient. Assuming a prevalence of MDR-TB of 3% among new cases and 17% among retreated cases, based on a DST survey conducted in Gujarat [12], a sample size of 123 confirmed new cases and 110 confirmed retreatment cases was sought in order to estimate the prevalence of MDR-TB, with 95% confidence intervals having a margin of error of 3% for new cases and 7% for retreated cases respectively.

### Study setting and study population

The study was carried out in five Mumbai District AIDS Control Society (MDACS) ART Centres [1) KEM Hospital, 2) SION Hospital, 3) SION Centre of Excellence in Paediatric HIV Care, 4) Godrej Hospital, Vikhroli and 5) Larsen & Toubro Hospital, Andheri] as well as in two Maharashtra State AIDS Control Society (MSACS) ART Centres [1) Thane Civil Hospital and 2) Navi Mumbai (Vashi) Municipal Corporation Hospital].

All HIV-infected adult and paediatric patients enrolled in the ART centres were potentially eligible to be enrolled in the study, if they had presumptive pulmonary or extra-pulmonary TB based on symptom screening, regardless of the time they were enrolled in the centres or whether they were on ART or not at the time of the study. Patients on TB treatment at the time of the study were excluded.

### Recruitment and sampling procedure

All HIV-infected ART center attendees were screened by an MSF-employed nurse during the study period. Patients with presumptive TB were investigated using a standard diagnostic algorithm recommended by the World Health Organization [13] that included TB culture and DST. The nurse explained in detail the objectives of the study to the patient and/or caregiver and obtained the signature or thumbprint of the patient if consent was given to participate. When pulmonary TB was presumed, two sputum specimens were collected on the same day, one hour apart, at each study site/hospital laboratory. When extra-pulmonary TB (EPTB) was presumed, biological specimens (fine needle aspirates, pleural fluid, cerebrospinal fluid, etc) were obtained from extra-pulmonary sites. All specimens were transferred to Hinduja Hospital Microbiology Laboratory in Mumbai for culture and first- and second-line DST.

Conventional microscopy with Ziehl-Neelsen (ZN) staining for acid-fast bacilli and further sputum decontamination was performed using the N-acetyl-L-cysteine and sodium hydroxide method. Concentrated sediment was inoculated in one liquid culture tube for testing using the Mycobacterial growth indicator tube (MGIT 960) method. Positive cultures underwent microscopy with ZN staining to confirm cord formation, and speciation with MPT 64 antigen detection by Immunochromatography was carried out to confirm *M. tuberculosis* complex. Specimens fulfilling the above criteria underwent further testing with phenotypic DST using the MGIT System for the following drugs: isoniazid, rifampicin, ethambutol, ofloxacin, moxifloxacin, kanamycin, capreomycin, PAS, ethionamide, clofazimine and linezolid. Non-tuberculous Mycobacteria (NTM) speciation was done by molecular methods using Reverse Line Blot Hybridisation. Hinduja laboratory is quality controlled and has been accredited for first-line DST by the WHO Supranational Reference Laboratory in Bangalore and the College of American Pathologists. The laboratory was also accredited by the TB programme for second-line DST in December 2013; prior to this date, if a strain was suspected to have resistance to one or more second-line anti-TB drugs, it was sent to the National Tuberculosis Institute Laboratory in Bangalore for confirmation.

Multidrug-resistant tuberculosis (MDR-TB) was defined as resistance to both isoniazid and rifampicin; pre-XDR-TB was defined as MDR-TB with additional resistance to either a fluoroquinolone or a second-line injectable agent; and extensively drug-resistant tuberculosis (XDR-TB) was defined as MDR-TB with additional resistance to both a fluoroquinolone and an injectable agent. Extremely drug-resistant tuberculosis (XXDR-TB) was defined as XDR-TB with additional resistance to any group IV and/or group V TB drugs (PAS, ethionamide, clofazimine, linezolid) [13].

### Management of those diagnosed with DR-TB

All patients diagnosed with MDR- or XDR-TB were managed in accordance with the national DR-TB treatment guidelines [14], while those with pre-XDR-TB were offered individualized treatment with 4 drugs likely to be effective.

### Data collection and analysis

Demographics, clinical and laboratory data, antiretroviral treatment (yes/no) and duration on ART, as well as data on previous TB treatment were doubly-entered into an EpiData database (Version 3.1, EpiData Association, Odense, Denmark), validated and analyzed.

To identify factors associated with TB and DR-TB, univariate and multivariate analyses were performed using Poisson and binary logistic regression models. Factors significant (p = 0.05) on univariate analysis were entered into the multivariate logistic regression models. Factors were coded as categorical variables and missing values for CD4 cell counts were imputed using a multiple imputation method. Transgender individuals (all were male to female) were grouped with biological males in the models. All factors were entered as a block into multivariate logistic regression models. Data analysis, including multivariate logistic regression models, was conducted with SPSS Version 20.0. Armonk, NY: IBM Corp. Released 2011).

## Results

**Figure 1:**
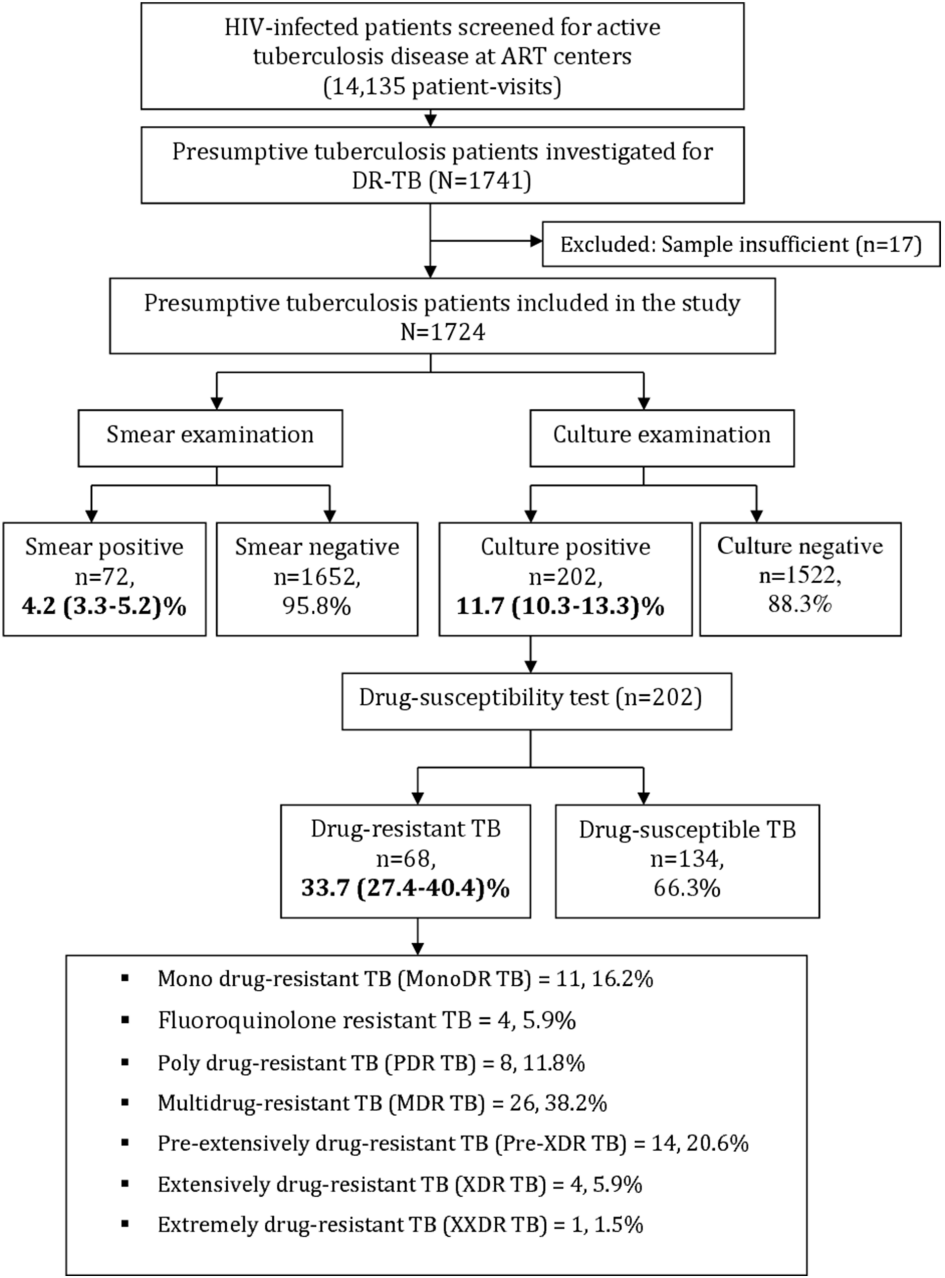
Drug-resistant tuberculosis among HIV-infected patients with presumptive tuberculosis, Mumbai, India.

Screening for presumptive TB was carried-out during 14,135 patient visits at seven ART centers in metropolitan Mumbai between March 2013 and January 2014 (Figure 1). Individual patients might have been screened more than once during the study period. A total of 1741 HIV-infected patients with presumptive tuberculosis (TB) were identified. All of them consented to participate in the study and were investigated for drug-resistant TB. The sputum specimens of 17 patients were found insufficient for laboratory investigations and had to be excluded. Thus, 1724 (99%) of the eligible patients were included in the study.

### Patient characteristics

The median age of the 1724 patients was 35 (Inter-quartile range, IQR: 24–44) years (Table 1) and the majority (60%) were male. A large proportion (53%) of patients had an average family income between 3500 and 7000 Indian National Rupees (equivalent to 60–120 USD) per month. Most of the patients (98%) had pulmonary TB. Among the entire study cohort, 80% were on ART during the study period and the majority (52%) had CD4 cell counts lower than 500 cells/µL at their last visit to an ART center. The median duration of exposure to ART prior to enrollment in the study was 26 months (IQR: 10.7–47.5). More than half (933/1724) of the presumptive TB patients had had at least one episode of active TB disease in the past.

**Table 1:** Demographic and clinical characteristics of HIV-infected patients with presumptive TB, Mumbai, India.

Characteristics	HIV-infected patients with presumptive TB (N = 1724)
	n (%)
**Age** [years, median (IQR)]	35.0 (24.3–44.0)
**Sex of patients**	
Male	1042 (60.4)
Female	671 (38.9)
Transgender	11 (0.6)
**Family income per month (in Rupees)**	
Less than 3500	88 (5.1)
3500–6999	910 (52.8)
7000 and above	454 (26.3)
Patient did not disclose	272 (15.6)
**TB site**	
Pulmonary	1688 (97.9)
Extra-pulmonary	36 (2.1)
**ART status**	
On ART	1386 (80.4)
Pre-ART	338 (19.6)
**CD4 count, last visit** (in cells/µl)	
Less than 200	258 (15.0)
200–349	351 (20.4)
350–499	289 (16.8)
500 and above	684 (39.7)
No information	142 (8.2)
ART duration* [months, median (IQR)]	26.0 (10.7–47.5)
**Previous episode of TB**	
Yes	933 (54.1)
No	791 (45.9)

ART: Antiretroviral treatment* Patients on ART with available information about ART initiation date, N = 1370.

### Culture-positive and drug-resistant tuberculosis

**Figure 2:**
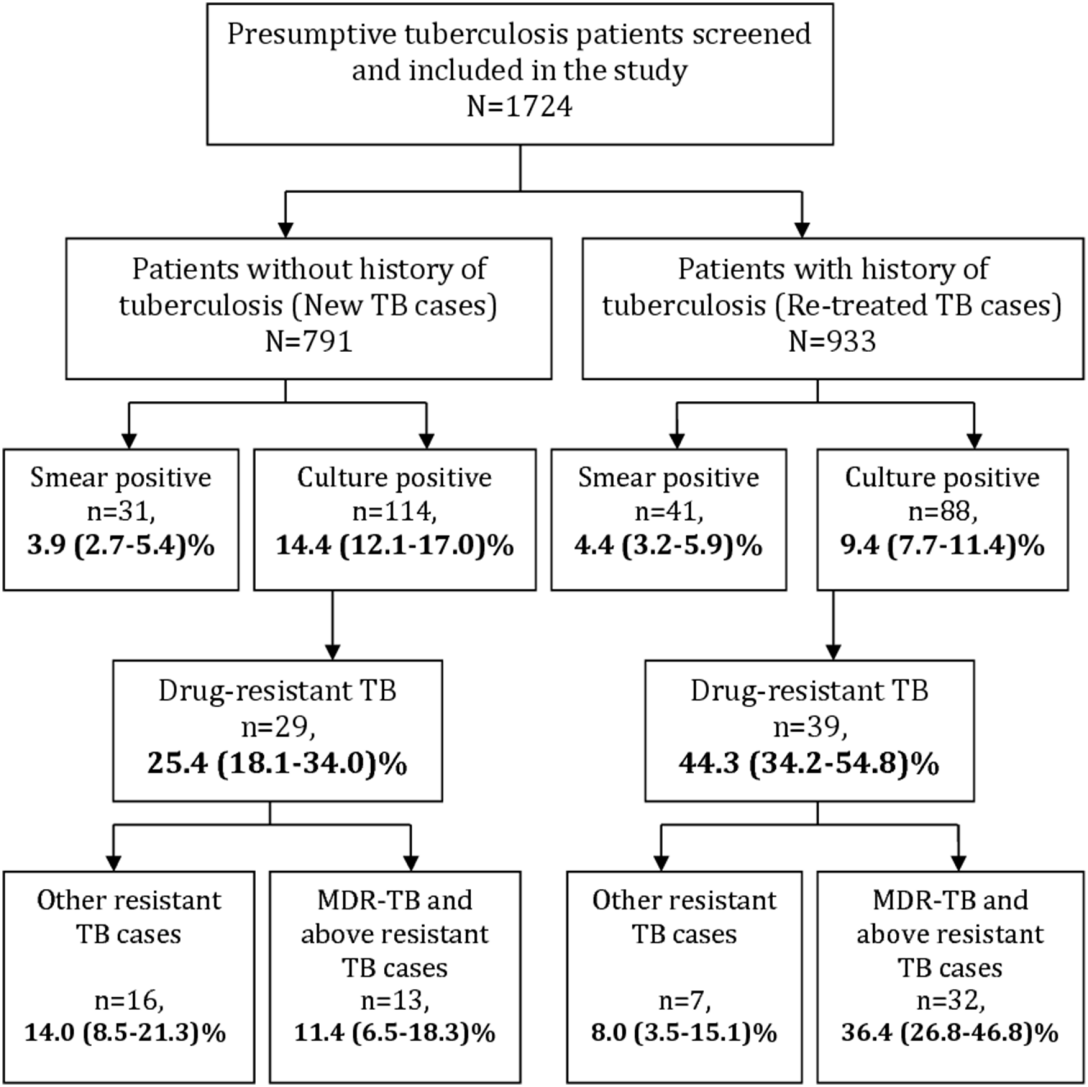
Distribution of Drug-resistant tuberculosis among HIV-infected (new and previously treated) with presumptive tuberculosis patients, Mumbai, India.

All of the 1724 patients with presumptive TB included in the study (Figure 1) underwent smear, culture and drug susceptibility testing (DST). Of these, 72 (4.2%; 95% Confidence Intervals (CI): 3.3–5.2) patients had smear-positive TB while 202 (11.7%; 95% CI: 10.3–13.3) patients had culture-positive TB. Eleven TB patients were smear-positive but culture negative and 141 patients were culture-positive but smear-negative. Those patients having a history of TB had a higher rate of smear-positivity (4.4% versus 3.9%), but lower culture-positivity rate (9.4% versus 14.4%) as compared to patients without TB history (Figure 2).

Among all culture-positive TB patients, 68 or 33.7% (95% CI: 27.4–40.4) had some form of drug-resistant TB. A high proportion of MDR-TB and pre-XDR-TB, 38% (26/68) and 21% (14/68) respectively, was observed amongst drug-resistant TB patients. Table 2 shows the detailed resistance patterns of all patients with DR-TB.

**Table 2:** Resistance profile (first and second-line) for all drug-resistant tuberculosis patients, Mumbai, India.

Resistance profile (culture-basedDST)	Total TBPatients(N = 68), n (%)	New TB patients(N = 29), n (%)	Previously treatedTB patients(N = 39), n (%)
H-mono	11 (16.2)	7 (24.1)	4 (10.3)
R-mono	-	-	-
Ofx-mono	1 (1.5)	1 (3.4)	-
Ofx Mfx	3 (4.4)	2 (6.9)	1 (2.6)
HE	7 (10.3)	6 (20.7)	1 (2.6)
HE Eto Ofx Mfx	1 (1.5)	-	1 (2.6)
HR	10 (14.7)	4 (13.8)	6 (15.4)
HRE	6 (8.8)	2 (6.9)	4 (10.3)
HR Eto	5 (7.4)	-	5 (12.8)
HR E Eto	5 (7.4)	2 (6.9)	3 (7.7)
HR Ofx Mfx E	2 (2.9)	2 (6.9)	-
HR Ofx Mfx Eto	1 (1.5)	1 (3.4)	-
HR Ofx Mfx E Lin	1 (1.5)	-	1 (2.6)
HR Ofx Mfx E Eto	8 (11.8)	1 (3.4)	7 (17.9)
HR Ofx Mfx E Eto PAS	2 (2.9)	-	2 (5.1)
HR Ofx Mfx Km Eto	1 (1.5)	-	1 (2.6)
HR Ofx Mfx Km E Eto	2 (2.9)	1 (3.4)	1 (2.6)
HR Ofx Mfx Km Cm E Eto	1 (1.5)	-	1 (2.6)
HR Ofx Mfx Km Cm E Eto PAS	1 (1.5)	-	1 (2.6)

H-isoniazid, R-rifampicin, E-ethambutol, Eto-ethionamide, Km-kanamycin, Cm-capreomycin, Ofx-ofloxacin, Mfx-Moxifloxacin, Lin- Linezolid, PAS- para-aminosalicylic acid.

Of the newly diagnosed (114/791) and previously treated (88/933) culture-positive TB patients, 25.4% (95% CI: 18.1–34.0) and 44.3% (95% CI: 34.2–54.8) patients had drug-resistant TB. The proportion of patients with multidrug-resistant TB and more advanced TB resistance profiles was higher (36% versus 11%) in previously treated patients compared to newly diagnosed TB patients.

### Children and extra-pulmonary tuberculosis patients

In the study, 283 children aged less than 15 years were investigated. The median (IQR) age of these children was 11 (8–13) years, just over half of them were male (56%), and sixty-eight percent were on ART. Of the 283 children investigated, 5% (15/283) had culture-positive TB, of whom seven (46.7%, 7/15) had drug-resistant TB; one had polydrug-resistant TB, two had MDR-TB and four had pre-XDR-TB.

Among 1724 HIV-infected patients investigated during the study period, 36 patients had presumptive extra-pulmonary TB (EPTB). The median (IQR) age of these patients was 45 (40–47) years and three-quarters of them were male (27/36, 75%). Among the 36 investigated presumptive EPTB patients, 14% (5/36) patients had culture-positive TB. Of these, 40% (2/5) patients had DR-TB: one had INH mono-resistant TB while another had pre-XDR-TB.

### Factors associated with culture-confirmed TB, DR-TB and MDR-TB

The demographic and clinical factors were assessed for association with culture-confirmed TB, DR-TB and MDR-TB. The univariate and bivariate analyses found age, ART status, CD4 count at last visit and previous episode of TB significantly related to culture-positive TB (Table 3). A multivariate Poisson regression model showed that older age, pre-ART status (i.e. not yet on ART), CD4 count less than 200 cells/µL at the last visit and a previous episode of TB were associated with culture-positive TB. None of the factors other than previous history of TB were associated with drug-resistant TB (Table 4) and multi-drug resistant TB (Table 5) in bivariate and multivariate binary logistic regression models.

**Table 3:** Demographic and clinical factors associated with culture-positive tuberculosis in HIV-infected patients, Mumbai, India.

Explanatory Variable	Patients withtuberculosis(N = 202), n (%)	Patientswithouttuberculosis(N = 1522), n (%)	Chi-square/t-test(p-value)	aPR^a^(95%CI)
**Age** [years, median (IQR)]	38.0 (32.0–43.3)	35.0 (22.0–44.0)	8.9 (<0.01)	0.99 (0.99–1.00)
**Sex of patients**				
Male	138 (13.1)	915 (86.9)	5.0 (0.02)	1.01 (0.99–1.03)
Female	64 (9.5)	607 (90.5)		
**Family income per month^+^** **(in Rupees)**				
Less than 5000	96 (10.9)	788 (89.1)	0.2 (0.65)	
5000 and above	66 (11.6)	502 (88.4)		
**ART status**				
Pre-ART	72 (21.3)	266 (78.7)	37.3 (<0.01)	1.07 (1.04–1.10)
On ART	130 (9.4)	1256 (90.6)		
CD4 count, last visit* (incells/µl)				
Less than 200	58 (22.5)	200 (77.5)	34.7 (<0.01)	1.08 (1.05–1.11)
200 and above	127 (9.6)	1197 (90.4)		
ART duration** [months,median (IQR)]	19.3 (5.2–35.7)	27.1 (11.3–47.9)	9.9 (<0.01)	
**Previous episode of TB**				
Yes	88 (9.4)	845 (90.6)	10.3 (<0.01)	1.01 (0.99–1.03)
No	114 (14.4)	677 (85.6)		

ART: Antiretroviral treatment, IQR: Inter-quartile range, CI; Confidence Intervals ^+^Patients with recorded family income, N = 1452* Patients with available information on CD4, last visit, N = 1582** Patients on ART with available information about ART initiation date, N = 1370^a^ aPR; adjusted Prevalence Ratios (calculated by Poisson regression using multiple imputation for CD4 missing data).

**Table 4:** Demographic and clinical factors associated with drug-resistant tuberculosis in HIV-infected tuberculosis patients, Mumbai, India.

ExplanatoryVariable	Patients withdrug-resistanttuberculosis(N = 68), n (%)	Patients withoutdrug-resistanttuberculosis(N = 134), n (%)	Chi-square/t-test(p-value)	aOR^a^(95% CI)
**Age** [years, median (IQR)]	35.5 (28.5–42.8)	38.0 (33.8–44.0)	2.15 (0.14)	0.98 (0.96–1.01)
**Sex of patients**				
Male	46 (33.3)	92 (66.7)	0.02 (0.88)	0.95 (0.49–1.82)
Female	22 (34.4)	42 (65.6)		
**Family income** **per month^+^ (in Rupees)**				
Less than 5000	36 (37.5)	60 (62.5)	1.8 (0.17)	
5000 and above	18 (27.3)	48 (72.7)		
**ART status**				
Pre-ART	22 (30.6)	50 (69.4)	0.48 (0.49)	0.96 (0.49–1.90)
On ART	46 (35.4)	84 (64.6)		
**CD4 count, last** visit* (in cells/µl)				
Less than 200	19 (32.8)	39 (67.2)	0.22 (0.88)	0.96 (0.48–1.93)
200 and above	43 (33.9)	84 (66.1)		
ART duration**[months, median(IQR)]	19.3 (5.7–31.3)	19.2 (3.2–43.7)		
**Previous episode** **of TB**				
Yes	39 (44.3)	49 (55.7)	7.93 (<0.01)	2.31 (1.24–4.30)
No	29 (25.4)	85 (74.6)		

ART: Antiretroviral treatment, CI; Confidence Intervals ^+^Patients with recorded family income, N = 162* Patients with available information about CD4 count, last visit N = 185** Patients on ART with available information about ART initiation date, N = 126^a^ aOR; adjusted Odds ratios (calculated by binary logistic regression using multiple imputation for CD4 missing data).

**Table 5:** Demographic and clinical factors associated with multidrug-resistant tuberculosis in HIV-infected tuberculosis patients, Mumbai, India.

ExplanatoryVariable	Patients withmultidrug-resistanttuberculosis (N = 45),n (%)	Patients withdrug-susceptibletuberculosis(N = 134), n (%)	Chi-square/t-test(p-value)	aOR^a^(95% CI)
**Age** [years,median (IQR)]	38.0 (30.0–42.5)	38.0 (33.8–44.0)	1.85 (0.18)	0.98 (0.95–1.01)
**Sex of patients**				
Male	30 (24.6)	92 (75.4)	0.06 (0.80)	0.83 (3.8–1.83)
Female	15 (26.3)	42 (73.7)		
**Family income** **per month^+^** **(in Rupees)**				
Less than 5000	21 (25.9)	60 (74.1)	1.03 (0.31)	
5000 and above	11 (18.6)	48 (81.4)		
**ART status**				
Pre-ART	12 (19.4)	50 (80.6)	1.69 (0.19)	0.85 (0.37–1.96)
On ART	33 (28.2)	84 (71.8)		
**CD4 count,** last visit*(in cells/µl)				
Less than 200	13 (25.0)	39 (75.0)	0.00 (1.00)	1.02 (0.43–2.41)
200 and above	28 (25.0)	84 (75.0)		
ART duration**[months, median(IQR)]	19.7 (5.7–34.8)	19.2 (3.2–43.7)	0.02 (0.88)	
**Previous episode** **of TB**				
Yes	32 (39.5)	49 (60.5)	16.2 (<0.01)	4.16 (1.93–8.95)
No	13 (13.2)	85 (86.7)		

ART: Antiretroviral treatment, CI; Confidence Intervals ^+^Patients with recorded family income, N = 140* Patients with available information about CD4 count, last visit N = 164** Patients on ART with available information about ART initiation date, N = 126^a^aOR; adjusted Odds ratios (calculated by binary logistic regression using multiple imputation for CD4 missing data).

## Discussion

To our knowledge this is the first DR-TB survey carried out among HIV clinic attendees in India. This study shows that, among HIV-infected children and adults in Mumbai, the burden of drug-resistant tuberculosis is extremely high: almost one in four new TB cases and one in two of those previously treated for TB have a drug-resistant strain. Of just as great concern, a large proportion of these strains was resistant to one or more second-line tuberculosis drugs, especially fluoroquinolones.

The overall rate of culture positivity amongst presumptive TB cases was surprisingly low (11.7%). We hypothesize that this was due neither to limitations in laboratory techniques nor the presence of NTM disease, but instead to the broad inclusion criteria that required a person attending a study site to have just one of four possible TB symptoms as recommended by WHO [13]; a person with ‘current cough’, for example, who was otherwise stable was eligible for enrolment. Another possible contributor to the low rate of TB culture positivity was the relatively large number of poor quality specimens (e.g. consisting of saliva) despite active instruction being given by a dedicated study nurse at each site. In any case, this finding warrants further investigation.

Even though the overall yield of TB was small in the pediatric cohort as well, it remains significant that almost half of the children with TB were infected with drug-resistant strains, most commonly pre-XDR-TB. Since bacteriological confirmation of DR-TB is more challenging in young children than in adults, as they cannot expectorate sputum and are more likely to have paucibacillary and extra-pulmonary TB, we hypothesize that the burden of TB and DR-TB is likely to be underestimated among children in this study, similar to what has been found in a recent meta-analysis [15]. With less than 2% of all study participants having specimens taken from extrapulmonary sites, it is almost certain that EPTB is being underdiagnosed as well in this cohort. A separate analysis found no significant association between EPTB and DR-TB in children or adults.

Our statistical models revealed no significant associations between most demographic and clinical factors and the risk of DR-TB and MDR-TB. We believe that these findings are important for their lack of associations; it seems that most TB/HIV co-infected patients attending ART centers in Mumbai are at risk for DR-TB. Although the relatively small sample size limits the power of our analyses and calls for cautious interpretation, the lack of associations suggests that all those infected with HIV and presumed to have active TB be tested for drug-resistant strains.

Given the high population density in Mumbai, in which a large proportion of the population lives in slums under extreme poverty, the very high TB prevalence and the relatively high HIV burden reported in greater metropolitan Mumbai, these data are unlikely to be representative of a country as vast and diverse as India. Nevertheless the living conditions in Mumbai and common practices in the public and private health sectors (as for example the prescribing of inappropriate regimens and over-the-counter availability of fluoroquinolones and other drugs with anti-TB properties) are similar to those of other large metropolitan centres in the country, so these data could very well represent the DR-TB situation in such cities as New Delhi, Kolkata and Chennai.

While it may not be possible to generalise our estimates for the entire country or even for HIV-uninfected populations, they serve to highlight the overall magnitude of the DR-TB epidemic in Mumbai, which is not unknown [16], [17]. A high prevalence of MDR-TB strains (11–68%) was reported in tertiary health facilities as early as 1991, followed by further documentation in 2006 [18]–[20], including information on the magnitude of the epidemic in children [21]. A study by D’Souza et al in 2009 [18] documented high levels of multiple drug resistance (both MDR and poly-drug resistance) amongst previously untreated cases in urban parts of Mumbai. In 2011 Udwadia et al reported a cases-series of totally-drug resistant TB (a term that has not officially been endorsed by WHO) in Mumbai, which captured the attention of local and international media [22], [23]. However to-date such findings are often overlooked and their importance minimized as representing only selected populations, laboratory or tertiary care settings and small case-series. Our study confirms that there is more than one epidemic ongoing in Mumbai and reinforces the urgent need to accurately measure the overall prevalence and incidence of DR-TB around the country in order to define appropriate interventions. Studies in selected populations such as this complement the overall estimates and can help in directing resources and prioritizing interventions targeted at the most vulnerable groups.

This survey is subject to the usual limitations in survey design and data collection. There is likely to be a tendency for patients to not report previous treatment either because they do not remember (recall limitation) or, on purpose, to avoid going through a long course of treatment that includes daily injectable medication and is known among patients for debilitating side effects [24]. Such bias could have led to an overestimate of DR-TB among new cases and an underestimate among retreatment cases. However, most HIV-infected patients attending ART clinics are usually aware of tuberculosis and have been counseled and screened for TB on several occasions, so recall limitation is rather unlikely.

The majority of HIV-infected patients attending public and public-private ART centers in the city are likely to access the public national TB programme for TB diagnosis and treatment. However many still seek care from private practitioners or may switch between the public and private sectors. The contribution to DR-TB levels from suboptimal treatment regimens prescribed in the unregulated Indian private health sector has been well documented [25]–[27]. Cox et al in 2007 have shown that even under well-established DOTS programmes in areas with high levels of drug resistance, high levels of amplification of drug resistance are to be expected [28].

The high level of resistance to three or more first-line anti-TB drugs and to fluoroquinolones has been previously described by others [29]. The proportion of previously untreated cases in our study that were resistant to more than three drugs, especially isoniazid, rifampicin and a fluoroquinolone, was particularly alarming and highlights two major issues in the management of TB in the setting of HIV/ART clinics. Firstly, it points to the scenario of nosocomial transmission of TB and DR-TB. Those attending an ART clinic at least once a month are more likely to be exposed to susceptible and resistant strains of *M. tuberculosis* than the general population. Given that the ART centers in Mumbai are usually extremely busy, constantly crowded and that they often lack adequate TB infection control interventions, this scenario is not unlikely. Instead of hypothesizing that most cases of DR-TB are due to non-adherence among patients on treatment, exogenous infection or re-infection should first be considered [30], [31]. Secondly, considering the high levels of resistance to second-line TB drugs and especially fluoroquinolones in this population, it is reasonable to assume that patients with presumptive TB may actually have pre-XDR-TB or even XDR-TB. This statement implies a huge investment in laboratory capacity in an already constrained public sector in Mumbai in order to screen all TB patients at the outset for strains that are resistant to fluoroquinolones and anti-TB injectables Nevertheless, we believe that it is a reasonable investment to make if the epidemic of DR-TB is to be controlled in the city in the future. Conversely, if DST is only offered afterward to those failing their TB treatment regimen, a large proportion of DR-TB cases will be missed due to the high risk of mortality among HIV-positive patients with untreated DR-TB [32].

There is an ongoing plan to systematically offer molecular TB diagnosis (mainly using Xpert MTB/RIF, also known as GeneXpert) to all HIV-infected patients in Mumbai and elsewhere in the country. While this is a giant leap forward, since GeneXpert can rapidly detect MTB and rifampicin resistance within 2 hours, we are concerned that ‘scale up’ of DR-TB diagnosis using this particular diagnostic may lead to suboptimal practices, since a diagnosis of rifampicin resistance alone and/or assumption that it represents a diagnosis of MDR-TB, may mask a diagnosis of pre-XDR or XDR-TB (or worse); the risks then associated with giving a suboptimal treatment regimen are significant both in terms of morbidity and mortality for the patient, as well as amplification of resistance and subsequent community transmission of resistant strains. While GeneXpert is an excellent and efficient diagnostic tool for MTB and screening test for DR-TB, in settings like Mumbai it is essential that it be complemented by culture and DST involving first- and second-line anti-TB drugs. The national programme has recently changed the policy to account for this risk, starting with HIV-infected patients in Mumbai and Maharashtra.

Our initial study protocol included fingerprinting studies using spoligotyping, which we had to abandon due to the high cost. Cox et al have in the past found a strong association between the Beijing genotype and amplification in situations of preexisting resistance in a central Asian setting [33]. Similarly, the proportion of the Beijing genotype was reported to be 35% in the urban Mumbai population studied by Almeida et al [34]. We need fingerprinting studies to establish how often nosocomial transmission occurs and to guide TB infection control interventions. Another area of research that is urgently needed relates to chemoprophylaxis for child contacts of DR-TB cases in Mumbai; preventative regimens that have shown to be effective in other settings are unlikely to prevent development of active disease in many children in Mumbai due to the high baseline rate of fluoroquinolone resistance [35].

## Conclusion

Our findings strongly suggest that there is an ongoing DR-TB epidemic among people living with HIV and attending ART centers in Mumbai, which requires urgent, innovative and feasible models of care that allow for rapid and accurate detection and treatment of as many DR-TB patients as possible. Ideally all patients with presumptive TB attending any ART center in Mumbai, or settings with similar drug resistance patterns, should be screened with a rapid molecular diagnostic followed by DST to first- and second-line anti-TB drugs, including for fluoroquinolones, so that the correct diagnosis is made as early as possible and followed by prompt treatment initiation with an appropriate individualized regimen. The high rate of DR-TB amongst new TB patients also highlights the need for better TB infection control measures in order to prevent ongoing transmission of DR-TB in the community and health facilities, especially those attended by vulnerable populations, such as those living with HIV.

## References

[1] World Health Organization (WHO) (2013), Global tuberculosis report 2013. WHO Press, Geneva, WHO/HTM/TB/2013.11.

[2] Department of AIDS Control (2013), National AIDS Control Organization, Annual Report 2012–2013, Ministry of Health & Family Welfare, Government of India.

[3] Paramasivan CN, Venkataraman P (2004) Drug resistance in tuberculosis in India. Indian J Med Res 120: 377–386. 15520487

[4] Deivanayagam CN, Rajasekaran S, Venkatesan R, Mahilmaran A, Ahmed PR, et al (2002) Prevalence of acquired MDR TB and HIV co-infection. Indian J Chest Dis Allied Sci 44: 237–242. 12437236

[5] Williams BG, Granich R, Chauhan LS, Dharmshaktu NS, Dye C (2005) The impact of HIV/AIDS on the control of tuberculosis in India. Proc Natl Acad Sci U S A 102: 9619–9624. 15976029 10.1073/pnas.0501615102 PMC1157104

[6] Swaminathan S, Paramasivan CN, Ponnuraja C, Iliayas S, Rajasekeran S (2005) Anti-tuberculosis drug resistance in patients with HIV and tuberculosis in South India. Int J Tuberc Lung Dis 9: 896–900. 16104637

[7] Maniar JK, Kanuth RR, Mandalia S, Shah K, Maniar A (2006) HIV and tuberculosis: partners in crime. Indian J Dermatol Venereol Leprol 72: 276–82. 16880573 10.4103/0378-6323.26723

[8] Pereira M, Tripathy S, Inamdar V, Ramesh K, Bhavsar M, et al (2005) Drug resistance pattern of Mycobacterium tuberculosis in seropositive and seronegative HIV-TB patients in Pune, India. Indian J Med Res 121: 235–239. 15817941

[9] Sethi S, Mewara A, Dhatwalia SK, Singh H, Yadav R, et al. (2013) Prevalence of multidrug resistance in *Mycobacterium tuberculosis* isolates from HIV seropositive and seronegative patients with pulmonary tuberculosis in north India. BMC Infect Dis 1471–2334/13/137. 10.1186/1471-2334-13-137 PMC3610146 23497169

[10] Menon S, Dharmshale S, Chande C, Gohil A, Lilani S, et al (2012) Drug resistance profiles of *Mycobacterium tuberculosis* isolates to first line anti-tuberculous drugs: a five years study. Lung India 29: 227–231. 22919160 10.4103/0970-2113.99104 PMC3424860

[11] Kumar P, Balooni V, Sharma BK, Kapil V, Sachdeva KS, et al (2014) High degree of multi-drug resistance and hetero-resistance in pulmonary TB patients from Punjab state of India. Tuberculosis (Edinb) 94(1): 73–80. 24184256 10.1016/j.tube.2013.10.001

[12] Ramachandran R, Nalini S, Chandrasekar V, Dave PV, Sanghvi AS, et al (2009) Surveillance of drug-resistant tuberculosis in the state of Gujarat, India Int J Tuberc Lung Dis. 13(9): 1154–1160. 19723407

[13] World Health Organization (2011) Guidelines for the programmatic management of drug-resistant tuberculosis. 2011 update. Geneva, Switzerland: WHO. 23844450

[14] Central TB Division (2012) Programmatic Management for Drug-resistant Tuberculosis guidelines-May version, Directorate General of Heath Services, Ministry of Health and Family Welfare. Available: http://www.tbcindia.nic.in/pdfs/Guidelines%20for%20PMDT%20in%20India%20-%20May%202012.pdf. Accessed 2014 May 5.

[15] Jenkins HE, Tolman AW, Yuen CM, Parr JB, Keshavjee S, et al (2014) Incidence of multidrug-resistant tuberculosis disease in children: systematic review and global estimates. Lancet 383(9928): 1572–1579. 24671080 10.1016/S0140-6736(14)60195-1 PMC4094366

[16] Almeida D, Rodrigues C, Udwadia ZF, Lalvani A, Gothi GD, et al (2003) Incidence of multidrug-resistant tuberculosis in urban and rural India and implications for prevention. Clin Infect Dis 36: e152–4. 12802779 10.1086/374931

[17] Singh S, Sankar MM, Gopinath K (2007) High rate of extensively drug-resistant tuberculosis in Indian AIDS patients. AIDS 21(17): 2345–7. 18090285 10.1097/QAD.0b013e3282f125c9

[18] D'souza DT, Mistry NF, Vira TS, Dholakia Y, Hoffner S, et al (2009) High levels of multidrug resistant tuberculosis in new and treatment-failure patients from the Revised National Tuberculosis Control Programme in an urban metropolis (Mumbai) in Western India. BMC Public Health 29: 9–211. 10.1186/1471-2458-9-211 PMC2714510 19563647

[19] Rodrigues C, Shenai S, Sadani M, Thakkar P, Sodha A, et al (2006) Multi drug-resistant tuberculosis in Mumbai: it's only getting worse. Int J Tuberc Lung Dis 10(12): 1421–1422. 17171830

[20] Chowgule RV, D Lina (1998) Pattern of secondary acquired drug resistance to antituberculosis drugs in Mumbai, India 1991–1995. Ind J Chest Dis Allied Sciences 40: 23–31. 9727280

[21] Karande S, Bavdekar SB (2002) Children and multidrug-resistant tuberculosis in Mumbai (Bombay), India. Emerg Infect Dis 8(11): 1360–1361. 12453371 10.3201/eid0811.020513 PMC2738557

[22] Udwadia ZF, Amale RA, Ajbani KK, Rodrigues C (2011) Totally Drug-Resistant Tuberculosis in India. Clin Infect Dis. doi:10.1093/cid/cir889. 10.1093/cid/cir889 22190562

[23] TIME Magazine (2013). Contagion; Why drug-resistant tuberculosis threatens us all. March 4, 2013.

[24] Isaakidis P, Rangan S, Pradhan A, Ladomirska J, Reid T, et al (2013) ‘I cry every day’: experiences of patients co-infected with HIV and multidrug-resistant tuberculosis. Trop Med & Int Health 18(9): 1128–1133. 23837468 10.1111/tmi.12146

[25] Uplekar M, Juvekar S, Morankar S, Rangan S, Nunn P (1998) Tuberculosis patients and practitioners in private clinics in India. Int J Tuberc Lung Dis 2(4): 324–329. 9559404

[26] Bhargava A, Pinto L, Pai M (2011) Mismanagement of tuberculosis in India: Causes, consequences, and the way forward. Hypothesis 9(1): e7.

[27] Udwadia ZF, Pinto LM, Uplekar MW (2010) Tuberculosis management by private practitioners in Mumbai, India: has anything changed in two decades? PLoS One 5: e12023. 20711502 10.1371/journal.pone.0012023 PMC2918510

[28] Cox HS, Niemann S, Ismailov G, Doshetov D, Orozco JD, et al.. (2007) Risk of acquired drug resistance during short-course directly observed treatment of tuberculosis in an area with high levels of drug resistance. Clin Infect Dis 441421–1427. 10.1086/517536 17479936

[29] Agrawal D, Udwadia ZF, Rodriguez C, Mehta A (2009) Increasing incidence of fluoroquinolone-resistant Mycobacterium tuberculosis in Mumbai, India. Int J Tuberc Lung Dis 13(1): 79–83. 19105883

[30] Andrews JR, Gandhi NR, Moodley P, Shah NS, Bohlken L, et al (2008) Exogenous reinfection as a cause of multidrug-resistant and extensively drug-resistant tuberculosis in rural South Africa. JID 198: 1582–1589. 18847372 10.1086/592991

[31] March F, Garriga X, Rodriguez P, Moreno C, Garrigo M, et al (1997) Acquired drug resistance in Mycobacterium tuberculosis isolates recovered from compliant patients with human immunodeficiency virus-associated tuberculosis. Clin Infect Dis 25: 1044–1047. 9402354 10.1086/516065

[32] Gandhi NR, Shah NS, Andrews JR, Vella V, Moll AP, et al (2010) HIV coinfection in multidrug- and extensively drug-resistant tuberculosis results in high early mortality. Am J Respir Crit Care Med 181: 80–86. 19833824 10.1164/rccm.200907-0989OC

[33] Cox HS, Kubica T, Doshetov D, Kebede Y, Rüsch-Gerdess S, et al (2005) The Beijing genotype and drug resistant tuberculosis in the Aral Sea region of Central Asia. Respiratory research 6(1): 134. 16277659 10.1186/1465-9921-6-134 PMC1299328

[34] Almeida D, Rodrigues C, Ashavaid TF, Lalvani A, Udwadia ZF, et al (2005) High incidence of the Beijing genotype among multidrug-resistant isolates of Mycobacterium tuberculosis in a tertiary care center in Mumbai, India. CID 40(6): 881–886. 10.1086/427940 15736024

[35] Seddon JA, Hesseling AC, Finlayson H, Fielding K, Cox H, et al.. (2013) Preventive therapy for child contacts of multidrug-resistant tuberculosis: a prospective cohort study. CIDcit655. 10.1093/cid/cit655 24065321

